# Dog10K: the International Consortium of Canine Genome Sequencing

**DOI:** 10.1093/nsr/nwz068

**Published:** 2019-05-29

**Authors:** Guo-Dong Wang, Greger Larson, Jeffrey M Kidd, Bridgett M vonHoldt, Elaine A Ostrander, Ya-Ping Zhang

**Affiliations:** 1 State Key Laboratory of Genetic Resources and Evolution, Kunming Institute of Zoology, Chinese Academy of Sciences, China; 2 Center for Excellence in Animal Evolution and Genetics, Chinese Academy of Sciences, China; 3 Palaeogenomics and Bio-Archaeology Research Network, School of Archaeology, University of Oxford, UK; 4 Department of Human Genetics and Department of Computational Medicine and Bioinformatics, University of Michigan Medical School, USA; 5 Ecology & Evolutionary Biology, Princeton University, USA; 6 National Human Genome Research Institute, National Institutes of Health, USA

Dogs (*Canis lupus familiaris*) were the first species to enter into a domestic relationship with people [1] and are a source of fascination all over the world, not only due to their history of domestication and dispersal along with human beings [2], but also because of their diverse phenotypes and behaviors, driven by both artificial and natural selection [3]. Dogs and humans have often been subjected to similar selection pressures [4], and these shared evolutionary trajectories have led to the emergence of the same common disorders including heart disease, neurologic disorders, diabetes and cancer [5].

The worldwide canine genetics genomics communities recently formed the International Consortium of Canine Genome Sequencing, also called the Dog10K Consortium (http://www.dog10kgenomes.org), to address major research questions regarding the genetic underpinnings of domestication, breed formation, aging, behavior and morphologic variation, and to advance our understanding of human and canine health [6]. This collaboration was originally conceived by the Chinese Academy of Sciences in 2016 with an initial plan to organize sample selection and collection, sequencing technology, bioinformatics, analysis and logistics, toward the goal of generating and sharing whole-genome sequence (WGS) data from 10 000 dogs and wild canids to improve the utility of the canine model system. An initial meeting was held in the summer of 2016 in the Academic Summit of Dog Genomes, featuring 25 participants representing 9 countries and 18 institutions (Fig. 1a), and the second meeting was held on 26 May 2019 at the 10th International Conference on Canine and Feline Genetics and Genomics (Fig. 1b) with 15 participants representing 13 institutions.

**Figure 1.**
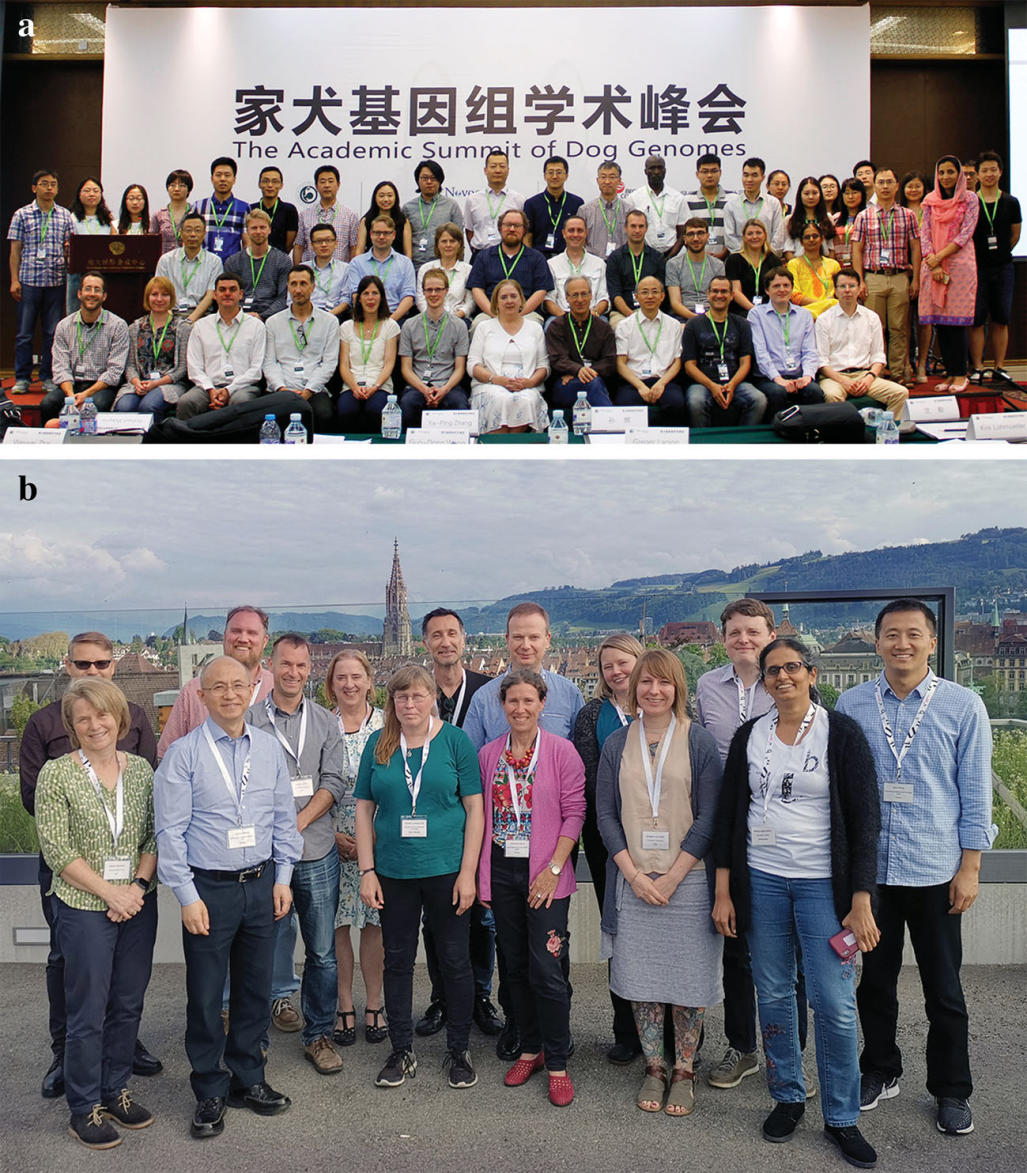
(a) Initial meeting of the Dog10K Consortium in Beijing, 2016 (*Courtesy of Yunyun Zheng*). (b) Second meeting of the Dog10k Consortium in Bern, 2019 (*Courtesy of Guo-Dong Wang*).

The primary goals of this collaborative endeavor are to generate WGSs of 10 000 canine/canids within 5 years, refine the existing reference genome from a Boxer, create new reference genomes from additional canids and apply the data to a myriad of scientific questions. The resulting catalog will contain comprehensive high-density genomic data, including single nucleotide variants (SNVs), structural variants (SVs), which include copy number variations (CNVs), and mobile element insertions (MEIs). Using these data, the possibility now exists to expand our knowledge of wild and domestic canids, and enhance studies of biology, disease susceptibility and evolution. We aim to sequence each genome to at least 20× coverage, which provides optimal coverage with minimal error rates. *De novo* genome assembly will be undertaken for both breed dogs and wild canids using a variety of technologies, including Pacific Biosciences long-reads (PacBio) (100×), Bacterial Artificial Chromosome (BAC)-end sequencing, optical mapping (Bionano Saphyr), phased haplotypes (60x by the 10x genomics platform), chromosome conformation (Hi-C), etc. We will sequence mRNAs, long non-coding RNAs and circle RNAs in pituitary, liver, hippocampus, ovarian and prefrontal cortex samples, and a full-length transcriptome through PacBio using a mix of the above tissues for the annotation of the reference genome. To address research questions, the Consortium defines the roles of participants and its interaction with the larger scientific community.

## Sample acquisition for phylogeny, domestication, and phenotypic and clinical traits

Integral to the success of Dog10K is the principle that sample collection will be extensive, covering all established breeds throughout the world, as well as village dogs, wild canids, and niche or non-registered dog populations that fall outside the formal definition of a recognized breed. The latter can be used as outgroups to improve detection of ancestral versus derived alleles. The wild canids and village dogs will help the demographic history and occurrence of admixture at the early stages of dog domestication to be deciphered. Niche population dogs will reveal the natural selection events that occurred when dogs traveled alongside humans during early human civilizations. Studies of established breeds will facilitate genetic studies of breed morphology, behavior and disease susceptibility. The Consortium is also sampling pedigrees spanning three generations for both dogs and wolves to generate deep, comprehensive genome sequence information, which will further enable an improved inference of evolutionary rates in canids.

## Bioinformatic analysis and data distribution

The Consortium will build a standard pipeline for SNV calling based on the Genome Analysis Toolkit (GATK) best practices [7] and will produce analysis-ready BAM files for each sample. Variant discovery will proceed from the BAM files and produce variant calls, which involves the generation of G.VCF files by the HaplotypeCaller for each sample and the production of variant calls by GenotypeGVCFs, combining all individuals. The output will be in VCF format for the further filtering of variants, refinement of genotypes and annotation. We will also use a combination of methods to call the SVs, CNVs and MEIs. Basic population genetic analysis will be performed to infer nuclear diversity, haplotypes recombination maps and other parameters.

Data sharing is a crucial component of the Consortium. The FASTQ, BAM and G.VCF files will be stored and shared through the Genome Sequence Archive (http://gsa.big.ac.cn/). FASTQ files will be stored and shared through the Sequence Read Archive (https://www.ncbi.nlm.nih.gov/sra/). At the same time, we will use iDog (http://bigd.big.ac.cn/idog/) to share omics data [8], [i.e. the data set of variations (SNVs, CNVs, SVs and MEIs), haplotype, gene expression, parameters of population genetics, etc.]. All data sets will be continuously updated throughout the duration of the project. Meanwhile, we will update the progress of the dog10K Consortium on the website (http://www.dog10kgenomes.org) and incorporate a variety of metadata, including information regarding the number of samples collected, gender, geography, progress to date and other sample-affiliated information. All cooperating organizations and related partners’ information will also be available on the website. For data generated by other means and incorporated into Dog10K, links will be established to the original websites where primary data and additional analyses are located.

## Population genomic- and WGS-based case-controls analysis

Dogs adapted to novel environments as they dispersed across continents with people and often hybridized with local wild canids as they did so. We will assess the strength and timing of selection at the first stage of dog domestication, and will identify artificial selection that affected early breeding processes. Dog10K data will allow for signatures of selection to be associated with specific traits and local adaptation processes in wild canid populations to be identified, both of which will serve as a basis for investigating the potential role of polygenic selection in canines, none of which have been studied to date. We will also explore the complex patterns of admixture among wild and domestic canid species, and search for genomic regions depleted of admixed ancestry and adapted introgression. Full genomes from diverse populations will provide new data to test ideas on the distribution of selection, which are integral to evolutionary models of demography and adaptation.

Strong artificial selection has resulted in ∼450 globally recognized breeds with distinct traits related to morphology, including body size, leg length, skull shape, etc. [9]. Dog10K will expand that applicability by developing an exhaustive catalog of genetic variation to enable the generation of an accurate imputation panel for dog genome-wide association studies, in much the same way that the 1000 Genomes Project has improved the study of low-frequency variants in human association studies [10,11]. Reference sequence data on all breeds will provide the scientific community with an ability to perform matched WGS-based case-controls analysis, either directly or through imputation, as has recently been successfully demonstrated for breed standard traits. The transition of the process to studies of disease will be permitted by this large data set. This aim is important, since the link between canine and human health has generated the most interest within the biomedical community. Advancing our understanding of common human diseases through studies of comparable diseases based on genomic approaches is key to the overall success of Dog10K.

## International collaboration

Dog10K aims to integrate quickly with ongoing sequencing and analysis efforts. For instance, the data set under construction includes the sequencing and subsequent analysis of 722 canids [9] and rare breeds from isolated locations throughout the world [12]. Other ongoing efforts in the general dog community fall into three categories. First, several research groups are studying domestication, independent of Dog10K [13,14]. The integration of WGS data from such studies will rapidly expand the utility of the Dog10K data set, permitting greater understanding of the selection events that have led to specific behaviors in domestic dogs as well as domestication itself [15]. Second, there are scientific programs focused on studies of ancient dog DNA (summarized in [16]). Such studies will provide insights into the links between wild canids and domesticated dogs, and will therefore be integrated into Dog10K. It is hoped that, simultaneously, the Dog10K reference data set will prove useful for studies of ancient canines. Finally, there are several ongoing efforts to build *de novo* reference genomes based on at least 12 distinct dog breeds or wild canids [17]. The aim of Dog10K is to produce an integrated reference genome with the caliber of the human genome. By both aiding the community with its advanced bioinformatics as well as directly integrating data produced by various research groups, this is sure to happen.

## CONCLUSION

The Dog10K Consortium will generate a comprehensive data set of unparalleled complexity and power. This effort will result in a resource that will prove invaluable to a wide range of fields, including comparative genomics, veterinary science, evolutionary biology, medical research and conservation genetics. Dog10K will thus elevate the dog to its rightful place as a premier genetic system for revealing fundamental insights into evolution, health and biology; therefore, Dog10K will be a valuable partner for the growing number of researchers who are investigating dogs as a viable model for studies of disease susceptibility, progression, treatment response and gene therapy outcomes [18,19].
